# Biochemical aspirin resistance is associated with increased stroke severity and infarct volumes in ischemic stroke patients

**DOI:** 10.18632/oncotarget.20356

**Published:** 2017-08-18

**Authors:** Xuan Cheng, Nan-Chang Xie, Hong-Liang Xu, Chen Chen, Ya-Jun Lian

**Affiliations:** ^1^ Department of Neurology, The first affiliated hospital of Zhengzhou University, Zhengzhou, Henan province, P.R. China

**Keywords:** aspirin resistance, infarct volume, stroke severity, diffusion-weighted imaging

## Abstract

To explore the correlation of aspirin resistance (AR) with clinical stroke severity and infarct volume using diffusion-weighted imaging (DWI) in 224 Chinese ischemic stroke patients who were taking aspirin before stroke onset. In those patients, the median age was 64 years (IQR, 56-75 years), and males accounting for 54.9%(123)of the total subjects. Fifty of 224 enrolled patients (22.3%; 95% confidence interval (CI), 16.9% to 27.7%) showed AR. In the median regression model, significant increase was estimated in NIHSS score of 0.04 point for every 1-point increase in aspirin reaction units (ARU) (95% CI, 0.02 to 0.06; *P*<0.001). Diffusion-weighted MRI (DWI)-measured infarct volume were significantly higher in patients with AR as compared with those with AS [13.21 (interquartile ranges [IQR], 8.51-23.88) *vs*.4.26 (IQR, 1.74-11.62); *P*<0.001). Furthermore, a statistically significant increase was also measured in NIHSS score of 0.05 point for every 1-point increase in ARU in the median regression model (95% CI, 0.03 to 0.08; *P*<0.001). The median DWI infarct volume was significantly larger in the highest ARU quartile when compared to that in the low 3 quartiles (P<0.001). In conclusion, stroke patients with AR indicated higher risk of severe strokes and large infarcts compared to patients in the aspirin-sensitive group.

## INTRODUCTION

Aspirin is the most frequently used anti-platelet agent for primary and secondary prevention of ischemic stroke [1]. It is generally considered that anti-platelet agents are the cornerstone of anti-platelet therapy in cardiovascular medicine, of which including aspirin in recent decades. By acethylating platelet cyclooxygenase, aspirin may exert the effect of anti-platelet, there by inhibiting platelet-dependent thromboxane formation irreversibly [2].

Previous evidence by incorporating large samples has documented that aspirin can result in the reduced incidence of both primary and secondary cardio-and cerebrovascular disease by approximately 25% [3]. However, aspirin cannot be effective in a great amount of patients who were diagnosed with myocardial infarction and stroke [4–5], which may be correlated with the phenomenon of aspirin resistance (AR). AR has received considerable attention recently, which is induced by the loss of efficacy of aspirin to reduce platelet production of thromboxane A2, which, in turn, causing platelet aggregation [6]. AR may contribute to negative impact on human beings, resulting in significant increase in health burden. However and crucially, pre-stroke use of anti-platelet agents possesses the opportunity of reducing the severity of initial stroke and correlated infarct volume [7–8]. However, with the administration of aspirin, an expected level of the inhibition of platelet is still estimated to detect in approximately 30% of stroke patients [9]. For example, Kim et al. [10] have documented in their study that with the inclusion of ischemic stroke patients at their acute stage, aspirin associated elevation in platelet reactivity might be related to early neurological deterioration independently over time. Meanwhile, previous evidence have suggested that elevated risk of recurrent stroke [11] and poor clinical outcomes [12] could be found in a number of stroke patients with AR.

Interestingly, a recent popular view is that there exist potential relationship between AR and the severity of stroke and corresponding infarct size [13–14]. The above issue, though, are still being debated, some of them held a supportive attitude [15–16], whereas others insisted the opposite [17–18].

In addition, infarct volume is regarded as an important surrogate of stroke outcome concerning it strong correlation with clinical stroke severity [19]. Nevertheless, the relationship between AR and infarct size by diffusion-weighted imaging (DWI) has not been assessed previously in Chinese stroke patients. The present study was thus conducted aimed at the exploration of the association of AR with clinical stroke severity and infarct volume using DWI in Chinese ischemic stroke patients who were taking aspirin before the onset of stroke.

## RESULTS

### Baseline characteristics of study samples

In this study, A total of336 patients were screened with acute ischemic stroke, finally 224 patients met the selection criteria and incorporated, including36 with onset of symptoms more than 24hours, 18 without informed consent, 8 without blood samples, 12 with malignant tumor,6 with renal insufficiency and44 unavailability of MRI results were not analyzed. In those patients, the median age was 64 years (IQR, 56-75 years), and 123 (54.9%) were men. Thirty-six out of the 224 patients (16.1%) were treated with tissue plasminogen activator-treated after admission. The score of raw ARUs ranged from 365 to 648, with a median value of 465 (IQR, 426-532). Of the enrolled patients, 50 patients were confirmed to have AR (22.3%; 95% CI, 16.9% to 27.7%), which was defined by above 550 ARU. The baseline characteristics of the 224 patients presenting with AR or AS were described in Table 1.

**Table 1:** Baseline characteristics of stroke patients with AR and AS

	Patients with AR	Patients with AS	P^a^
N (%)	50(22.3)	174(77.7)	<0.001
Age, years medians (IQRs)	65(57-77)	63(56-74)	0.632
Sex, male/female, n	29/21	94/80	0.618
BMI, kg/m2 medians (IQRs)	26.8(25.2-28.1)	26.7(25.4-28.2)	0.402
SBP, mmHg medians (IQRs)	142(120-155)	136(115-150)	0.198
Vascular risk factors, n (%)			
Hypertension	36(72.0)	118(67.8)	0.574
Diabetes	22(44.0)	60(34.5)	0.218
Coronary heart disease	11(22.0)	32(18.4)	0.631
Hypercholesterolemia	19(38.0)	61(35.1)	0.775
History of TIA	15(30.0)	34(19.5)	0.115
Smoking habit	17(34.0)	59(33.9)	0.942
Pre-stroke treatment, n (%)			
Antihypertensive drug	32(64.0)	84(48.3)	0.001
Statin use	15(30.0)	62(35.6)	0.336
Administration of IV-tPA, n (%)	10(20.0)	32(18.4)	0.803
Median onset to ARU, (hr, IQR)	20.2(14.3-24.2)	17.5(13.2-22.8)	0.205
Median onset to DWI, (hr, IQR)	15.4(11.0-16.8)	13.2(9.5-14.7)	0.368
Stroke etiology no. (%)			0.713
Cardioembolic	9(18.0)	32(18.4)	
Small-vessel disease	11(22.0)	43(24.7)	
Large-vessel atherosclerosis	17(34.0)	66(37.9)	
Other	7(14.0)	17(9.8)	
Unknown	6(12.0)	16(9.2)	
Stroke syndrome no. (%)			0.016
Embolic strokes (TACS, PACS, and POCS)	31(62.0)	137(78.8)	
Arteriosclerotic strokes(LACS)	19(38.0)	37(21.2)	
NIHSS at admission, medians (IQR)	8(5-15)	3(1-6)	<0.001
Lesion volumes, median(ml, IQR)	13.21(8.51-23.88)	4.26(1.74-11.62)	<0.001
Laboratory data, median (IQR)			
ARU	585(566-613)	450(410-477)	<0.001
WBC, x109/L	8.8(7.2-9.8)	7.6(6.5-9.0)	0.021
Platelet, X10^3^/ml	245(210-273)	238(206-270)	0.556
FBG, mmol/l	5.77(4.89-6.90)	5.21(4.55-6.42)	0.032
CRP, mg/l	5.68(3.99-8.65)	3.22(2.04-5.11)	0.003
Total cholesterol, mg/dl	199(134-255)	190(130-247)	0.572
LDL cholesterol, mg/dl	128(101-145)	125(98-140)	0.637

^a^*p* value was assessed using Mann-Whitney U test or Chi-Square test.

AR, aspirin-resistant; AS, aspirin-sensitive; IQR, interquartile range; NIHSS, National Institutes of Health Stroke Scale; TPA-T: Tissue plasminogen activator-treated; CRP, C-reactive protein; FBG, fasting blood glucose; SBP, Systolic blood pressure; LACS, lacunar syndrome; PACS, partial anterior circulation syndrome; POCS, posterior circulation syndrome; TACS, total anterior circulation syndrome; BMI, body mass index; ARU, aspirin reaction units; DWI, Diffusion-Weighted Magnetic Resonance Imaging

By comparison, patients with embolic strokes (TACS, PACS, and POCS, n= 168) had lower ARU than those with intrinsic arteriosclerotic strokes (LACS, n=56) [450(412-511) vs. 489(IQR: 440-556); P= 0.022]. In addition, stroke patients with antihypertensive drug had higher ARU than those without administration [505(IQR: 463-588) vs. 425(398-493); P=0.004]. Furthermore, obvious positive correlations were measured between ARU and WBC (r=0.186, *P*=0.021), FBG(r=0.206; P=0.032) and CRP(r=0.232, P=0.003). However, in the comparison of other baseline data, no statistical differences were found between AR group and AS group in age, BMI, blood pressure, smoking status, stroke etiology, pre-stroke and acute treatment, time to MRI and blood collection.

### AR and the severity of stroke

**Figure 1:**
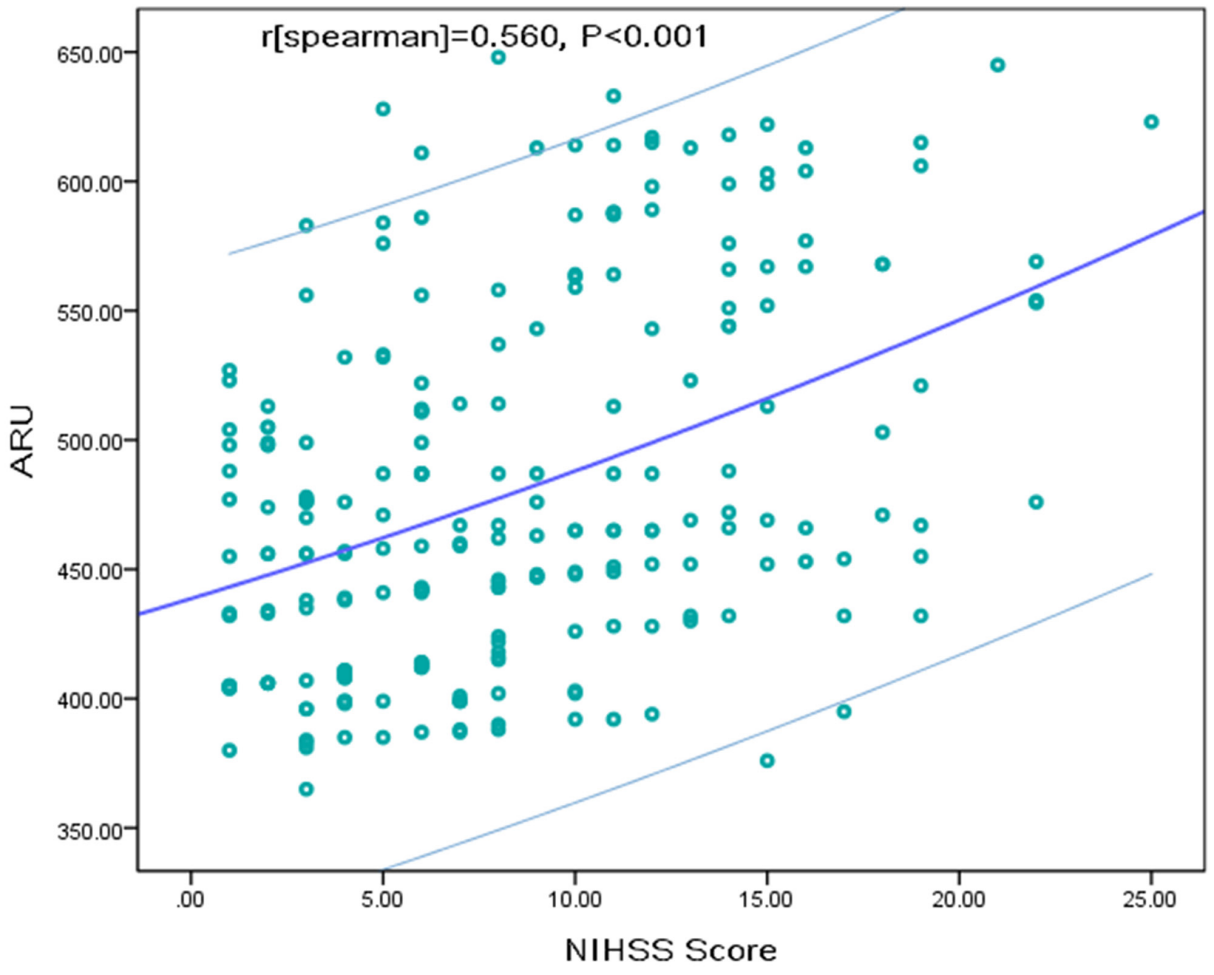
The linear association between ARU and NIHSS score The slash lines represent the trend line and 95%CI. ARU=aspirin reaction units; NIHSS=National Institutes of Health Stroke Scale.

Spearman rank correlation indicated that NIHSS was positively correlated with raw ARU scores (r=0.560, *P*<0.001; Figure 1), the significance was also existed with the adjustment for vascular risk factors. In the median regression model, NIHSS score of 0.04 point was measured to be increased significantly for every 1-point increase in ARU (95% CI, 0.02 to 0.06; *P*<0.001), amount to a median increase of about 1 point in NIHSS score for every 25-point increase in ARU. Meanwhile, AR was evidently related to the severity of stroke (Table 1). Observed median NIHSS score in the AR group was remarkably higher than that in the AS group [8 (IQR, 5-15) vs.3 (IQR, 1-6)], with an obvious median difference of 5 by statistical analysis (95% CI, 3.17 to 8.16; P<0.001). In addition, AR patients were could sustain a moderate-to-high severe stroke, which was determined with NIHSS score of over 6 points (OR, 6.06; 95%CI, 2.29-16.02; P = <0.001). After adjustment of age, sex, and other risk factors, multivariable model analysis revealed that AR was still associated with an increased risk with NIHSS>6 (OR=4.55; 95% CI = 2.42–9.18;P<0.001), which was described in Table 2. In those no-cardioembolic strokes (N=183), AR was related to NIHSS score(r=0.512, *P*<0.001), and also associated positively with higher risk of a NIHSS>6 (OR=4.32; 95% CI = 2.23–8.53; P<0.001).

**Table 2:** Multivariate analysis of predictors of moderate-to-high clinical severity ^a^

Predictors	OR	95% CI	P
AR *vs*. AS	4.55	2.42-9.18	<0.001
Age (per unit increase)	1.22	1.11-1.39	0.026
Infarct volume (per unit increase)	1.17	1.10-1.33	0.009
Stroke etiology (lacunar subtype)	0.48	0.35-0.59	<0.001
FBG(per unit increase)	1.24	1.10-1.45	0.016
CRP(per unit increase)	1.35	1.20-1.77	0.018

^a^ Multivariable model included all of the following variables: age, gender, BMI, stroke syndrome, stroke etiology, vascular risk factors, acute and pre-stroke treatment, lesion volumes, and blood levels of WBC, Platelet, Total cholesterol, LDL cholesterol, CRP and FBG; moderate-to-high clinical severity is defined as NIHSS score >6.

OR, odds ratio; CI, confidence interval; FBG, fasting blood glucose; CRP, C-reactive protein; NIHSS, National Institutes of Health Stroke Scale; AR, aspirin-resistant; AS, aspirin-sensitive

### AR and DWI infarct volume

**Figure 2:**
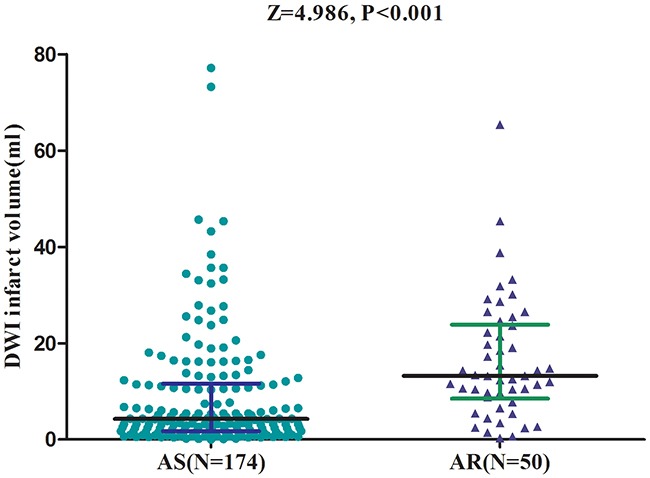
Variation of DWI Infarct volume between AS and AR patients All data are medians and in-terquartile ranges (IQR). *P* values refer to Mann-Whitney *U* tests for differences between groups. AR=aspirin-resistant; AS=aspirin-sensitive; DWI=Diffusion-Weighted Magnetic Resonance Imaging; ARU=aspirin reaction units

Median DWI infarct volume was 5.43mL (IQR, 2.20-14.39mL) in all patients. DWI infarct volume was much higher in patients with AR than those with AS [13.21 (IQR, 8.51-23.88) *vs*.4.26 (IQR, 1.74-11.62); Z=4.986, *P*<0.001; Figure 2). The median DWI infarct volumes for the four quartiles of ARU were 2.79, 4.03, 4.92 and 13.12ml from the lowest to the highest, respectively (Table 3). DWI infarct volumes indicated a positive skewed distribution, reflecting a larger proportion of patients with smaller infarct volumes (skewness, 5.5; kurtosis, 16.2). Furthermore, positive correlation was also detected between ARU and DWI infarct volume (*r*=0.631; *P*<0.001) before and after the adjustment for vascular risk factors. At the same time, increased NIHSS score of 0.05 point was also observed for every 1-point increase in ARU (95% CI, 0.03 to 0.08; *P*<0.001), amount to a median increase of NIHSS score of about 1 point for every 20-point increase in ARU. In the 183 patients with no-cardioembolic strokes, positive correlation between ARU and DWI infarct volume was also observed by applying nonparametric spearman rank correlation analysis (*r*=0.601; *P*<0.001).

**Table 3:** Relationship between ARU quartile and DWI infarct volume based on multivariate quartile regression analysis

ARU at admission	Median Infarct Volume(ml, IQR)^a^	Multivariate Change in Infarct Volume (95% CI),mL^a, b^	P value ^c^
Q1(<426)	2.79(0.98-7.45)	Reference(1.00)	—
Q2(426-465)	4.03(1.72-9.37)	1.15(0.95-2.53)	0.105
Q3(466-532)	4.92(2.12-12.99)	1.92(1.31-3.16)	0.021
Q4(>532)	13.12(6.82-25.12)	5.44(3.05-10.21)	<0.001

^a^ *P<0*.001 for trend across quartiles

^b^ adjusted for age, sex, BMI, stroke subtype, stroke syndrome, vascular risk factors, prior or acute treatment, Time from onset to ARU, time to MR imaging, NIHSS score and blood levels of WBC, Platelet, Total cholesterol, LDL cholesterol, CRP and FBG.

^c^ Post hoc test of difference in DWI infarct volumes compared with reference Q1.

DWI, Diffusion-Weighted Magnetic Resonance Imaging; CRP, high C-reactive protein; FBG, fasting blood glucose; IQR, interquartile range; NIHSS, National Institutes of Health Stroke Scale; ARU, aspirin reaction units

Table 3 presented results of univariate and multivariate analyses ARU and pretreatment DWI infarct volume. Univariate analysis showed the statistically significant quartile regression model for relationship exploration, suggesting positive trend between ARU and median DWI infarct volume (P<0.001). Meanwhile, compared to the data in the lowest ARU quartiles, median DWI infarct volume was significantly larger in the highest ARU quartile (P<0.001). Multivariate analysis proved once again the overall trend toward reduced DWI infarct volumes across ARU quartiles significantly (P<0.001; lowest to highest). Further, after adjustment for other factors, median DWI infarct volumes (IQR) were2.45, 3.83, 4.45, and 9.47ml for each of the ARU level quartiles from lowest to highest quartiles, respectively, which were indicated in Table 3.

## DISCUSSION

Previous studies had suggested the significance of prior use of aspirin for lowering NIHSS scores [20] as well as decreasing modified Rankin Scale scores [21] in patients in hospitalization and discharging, respectively. At the same time, infarct growth was also reduced by prior anti-platelet therapy in human studies [22]. Results in the current research suggested that the patients with AR had a higher median infarct volume using DWI compared with those with AS, indicating larger infarct size; the former patients were also showed to combine with worse neurological deficit. The study is the first study with novel findings in Chinese population. According to the criteria of NIHSS and the determination of infarct size, higher levels of ARU may indicate worse neurological deficit. Similarly, Zheng et al. [13] also demonstrated the positive association between AR and the severity and infarct volume in stroke patients at their acute phase, which was also the same in patients taking aspirin before the onset of stroke [14]. Further, AR could predict new ischemic lesions during the follow-up period by examining DWI in stroke [23].

It was estimated that AR may occur in 5%-65% patients with ischemic strokes [24]. In the study, the percentage of AR by taking aspirin (22.3%) was in accordance to that reported from a recent meta-analysis (23%, 95%CI: 20-28%) [25]. In addition, the incidence of AR in patients with stroke was 16.3%in the study by Kim et al. [23], while it was 30% in another study [9]. Furthermore, Berrouschot et al. [26] reported that in the enrolled 291 patients, 7.2% and 4.1% were identified as primary and secondary ASA-non-responders initially and during the period of follow-up, respectively. The observed prevalence of AR was relatively wide in range, which might correlated with the cause of the different tests to examine the platelet function, as well as the differences in baseline characteristics and clinical data. Interestingly, In addition, it was suggested that AR was more common in patients with lacunar strokes than those with embolic strokes [18, 27], which was also identified in the present study (33.9% vs. 18.4%, p=0.016).

There were multiple studies focused on exploring the association of AR with infarct size and stroke severity [13–14, 18, 28]. To be specific, Zheng et al. [13] found that AR might indicate more extensive tissue injury, similar to that in other study [14], yet none apparent association between them was found by El-Mitwalli et al. [18]. Similarly, another study exhibited no obvious difference among AS and AR patients considering the NIHSS score or DWI lesion volume on admission [28]. In this study, AR was positively related to increased severity and infarct volume in Chinese subjects with acute stroke. Therefore, we hypothesized that the disparate results of our study and those in the previous research could be correlated with the insufficient number of study participants, methodologic differences, and different imaging technique to assess early ischemic change, diverse measurement time points, heterogeneous stroke subtypes, and clinically imprecise neurologic scales, etc. [14].

Mechanisms associated with the phenomenon that AR increases the severity and infarct size of stroke have been investigated by multiple researchers. First, in an animal model, carotid mural thrombi was significantly decreased by pretreatment with aspirin [29]. Theoretically, platelet aggregation inhibitors are developed to inhibit the induced platelet aggregation and play an antithrombotic role. In this regard, insufficient inhibition of platelet aggregation in patients with AR may lead to a larger thrombus and accumulating microvascular thrombi, in turn conducive to more severe degree of stroke and larger infarct volumes [30]. Conversely, AR is a common phenomenon occurred in patients with the administration of aspirin like other anti-platelet drugs. AR may represent the burden of atherosclerotic disease. In terms of relevant mechanism, it was postulated that inadequate platelet inhibition induced larger thrombus formation responsible for the increased severity and infarct size. Second, in view of the function of aspirin, it is prepared as an anti-inflammatory and neuroprotective agent, which may therefore possess the effect of reducing the severity of stroke [31]. In some cases, larger areas of ischemic tissues may produce more profound prothrombotic inflammatory reactions and may thereby contribute to AR-platelet aggregation during the acute phase of atherosclerotic disease [32–33]. Third, aspirin can also exert the role of reducing platelet microaggregates and platelet derived vasoconstricting products [34]. The same therapeutic effect of aspirin was failed in patients with AR and, as a result, those patients might sustain a higher risk of stroke. Although our study design does not allow us to make conclusions about cause and effect relationships between AR and stroke severity, findings in the study extend knowledge of the relationship between AR and stroke severity. Interestingly, on the basis of the severity of thrombosis, a previous study also made an assumption that the effects of AR might differ across the conditional percentiles of initial NIHSS scores and DWI infarct volumes [14].

### Strengths and limitations

Firstly, the most important strengths in the study were the extensive measurement of covariates, the rigorous adjustment for variables which were proved to be related to ischemic lesion volume, as well as a sensitive imaging technique (DWI) usage, and blinded imaging assessment. Our study demonstrated a distinct association between AR and infarct volume, which indicates brain tissue with failing cellular energy-dependent processes during early ischemic stroke. Thus, compared to initial CT, DWI is a more refined method for assessing changes in early ischemic tissue. In addition, early DWI infarct volumes strongly correlate with final infarct volumes on T2-weighted imaging [35]. Further, a different strategy using the fourth quartiles was selected in the study, resulting incomprehensive understanding of the effect of AR on the severity and infarct volume of stroke deeply and widely.

Some limitations in this study should be considered. First, the included stroke patients in the study received imaging before any reperfusion therapy within 24 hours of hospitalization. However, the obtained data on day 1 are not considered to be a good measure of the completed stroke volume. In addition, infarct volume was calculated in a non-accurate way, and there was no further evaluation with respect to numbers and location of the infarct. Future studies on location of the infarct and white matter changes will be needed to further disentangle the effect of these factors on outcomes. Those factors will cause measurement bias. Second, whether AR is pathogenically involved in the development of infarct volume would be a more interesting topic, nevertheless, the observational study does not allow advancing any cause and effect relationships. In addition, as an observational protocol with all inherent limitations, potential unmeasured confounders could be present accordingly. Third, only one platelet function test was involved to assess AR in patients at admission. Additional laboratory tests that detect plateletcyclooxygenase-1 function should be performed to verify the aspirin compliance and aspirin responsiveness [36]. Any way, it should be noted that VerifyNow assay is a simple and rapid point-of-care test for assessing AR, which is readily accessible in clinical practice, which also confirm the clinical significance of findings drawn from our study on the side [14]. Fourth, all involved patients were diagnosed as acute stroke, the AR values and infarct volume were only measured at the acute stage of stroke. Further study about another time-point AR value and infarct volume can help elucidate the association of AR and stroke. Lastly, this was a single hospital-based observational study with relatively smaller sample size; unmeasured confounding factors are therefore uncontrollable. Accordingly, our findings should be interpreted with caution given the above limitations when generalizing to other patients with ischemic stroke.

## MATERIALS AND METHODS

### Ethical statement

An approval of the study was got from the ethics committee of the Affiliated Hospital of Zhengzhou University. All participants or their relatives were informed about the study protocol, and their written informed consents were obtained before participating in the study.

### Patients

Consecutive patients with first-episode of ischemic stroke were identified in the study, who was admitted to the Emergency Department of our hospital from January 2014 to December 2016. Inclusion criteria were pre-set: (1) on aspirin therapy regimens (acetylsalicylic acid, 100 mg daily) for over 1 week prior to the onset of stroke; (2) ischemic infarct was determined by magnetic resonance imaging (MRI); (3) a new focal or global neurological event within 24 hours; (4) with informed consents signed and provided before the performance of the study. The determination of patients’ compliance was achieved by interviewing patients and their relatives. Patients were excluded from the study due to medical history of malignant tumor, renal insufficiency (creatinine >1.5 mg/dl), severe edema, lost blood samples, history of brain trauma and cerebrovascular disease (CVD) in past 3 months, as well as past platelet function disorders, or concurrently administration history of an additional anti-platelet, anticoagulant, or non steroidal anti-inflammatory medication.

Demographic data were recorded and collected in the initial stage of the experiment, covering two major categories, baseline characteristics of age, sex, and body mass index (BMI) and vascular risk factors, such as blood pressure, smoking habit, hypertension, diabetes mellitus, hypercholesterolemia, coronary heart disease and a history of transient ischemic attack (TIA). Meanwhile, patients who received pre-stroke therapy (antihypertensive and/or statins) and acute treatment (Tissue plasminogen activator-treated [TPA-T]) were also recorded. Stroke severity was determined in each patient on admission by a neurologist (Cheng X). The National Institutes of Health Stroke Scale (NIHSS) was the single judgment standard in the study for strokes severity. Furthermore, the classification of stroke was achieved by using Trial of Org 10172 in Acute Stroke Treatment classification (TOAST) and the clinical stroke syndrome was determined applying Oxfordshire Community Stroke Project (OCSP).

Before any therapy, patients within 24 hours on admission had their imaging examination using a Siemens Vision 3.0-T scanner (Siemens Medical Systems, Erlangen, Germany). Infarct volumes indicated by DWI were measured with version 3.0MIPAV software (NIH, Bethesda, MD) [37]. With the application of a semiautomatic segmentation method, acute diffusion lesions were identified on a slice-by-slice basis, meanwhile, apparent diffusion coefficients were taken into consideration to distinguish acute from non-acute diffusion signals. DWI infarct volumes were calculated by multiplying slice thickness by total areas of lesions.

Fasting blood samples were collected from each patients on the first morning after admission (within 0–3 [n=24], 3–12 [n=76], 12–24 [n=85], and 24–48 [n=39] hours from symptom onset). Ultegra Rapid Platelet Function Assay-ASA (VerifyNow aspirin, Accumetrics, San Diego, California) was applied for the measurement of aspirin induced platelet inhibition. The result was expressed in aspirin reaction units (ARU), with a time interval of 5 min for each blood sample. The cut-off point was set as 550 ARU in accordance with protocol of the manufacturer using optical aggregometry as the comparison standard. A cutoff of 550 ARU determined the presence of AR [13], with the value ≥550 IU as AR, while <550IU as aspirin sensitive (AS). Raw ARU scores were presented as continuous variables for determining the extent of platelet aggregation [38]. Furthermore, C-reactive protein (CRP), fasting blood glucose (FBG), platelet, total cholesterol, LDL cholesterol and white blood count (WBC) were also measured using routine laboratory methods.

Data such as categorical variables were expressed as percentages and continuous variables were presented as medians (interquartile ranges, IQRs). Comparisons between groups were conducted using Mann-Whitney U test and Chi-square test. Spearman's rank correlation test was used for correlation exploration of laboratory parameters. Logistic regression model was constructed to identify the associations between AR and stroke severity (dichotomized as NIHSS ≤5 and NIHSS>6) [39], and DWI infarct volume. Semi-parametric univariate and multivariate quartile regression analyses were included to identify the association between median DWI infarct volume and ARU level quartiles [40]. Median DWI infarct volumes were adjusted for potential confounding variables in the multivariate model. Covariates included age, sex, BMI, stroke subtype, stroke syndrome, vascular risk factors, prior or acute treatment, time from onset to ARU, time to MR imaging, NIHSS score and blood levels of WBC, platelet, total cholesterol, LDL cholesterol, CRP and FBG. Results related to the regression analyses were expressed as adjusted odds ratios (OR) with95% Confidence interval (CI). SPSS 22.0 (SPSS Inc., Chicago, IL, USA) was used for all statistical analyses, P<0.05 indicated the existence of statistical difference during the process of comparison.

## CONCLUSIONS

In conclusion, stroke patients with AR might have severe strokes and large infarct volumes compared to patients with AS. Simultaneously, randomized controlled study is recommended for the comparison of the effect of alternative anti-platelet agents in patients with AR. Laboratory tests for AR should also be considered before modifying an anti-platelet regimen in stroke patients who taking aspirin.

## Abbreviations

AR: Aspirin resistance
DWI: Diffusion-weighted imaging
CVD: cerebrovascular disease
NIHSS: National Institutes of Health Stroke Scale
MRI: Magnetic Resonance Imaging
TIA: Transient ischemic attack
BMI: Body mass index
TPA-T: Tissue plasminogen activator-treated
OCSP: Oxfordshire Community Stroke Project
MIPAV: Medical Image Processing Analysis and Visualization
ARU: Aspirin reaction units
AS: Aspirin sensitive
WBC: White blood cell count
CRP: C-reactive protein
FBG: Fasting blood glucose
IQR: Interquartile range
CV: Coefficients of variation
ORs: odds ratios
CI: Confidence interval
